# An automated home-cage-based 5-choice serial reaction time task for rapid assessment of attention and impulsivity in rats

**DOI:** 10.1007/s00213-019-05189-0

**Published:** 2019-03-02

**Authors:** B. Bruinsma, H. Terra, S. F. de Kloet, A. Luchicchi, A. J. Timmerman, E. Remmelink, M. Loos, Tommy Pattij, Huibert D. Mansvelder

**Affiliations:** 1 Department of Integrative Neurophysiology, Center for Neurogenomics and Cognitive Research (CNCR), Amsterdam Neuroscience, VU University, Amsterdam, The Netherlands; 2 Department of Anatomy and Neurosciences, Amsterdam Neuroscience, VU University Medical Center, Amsterdam, The Netherlands; 3 Sylics (Synaptologics B.V.), Amsterdam, The Netherlands

**Keywords:** 5-CSRTT, Rats, Home-cage, Attention, Impulsivity, Scopolamine, Animal model

## Abstract

**Rationale:**

The 5-choice serial reaction time task (5-CSRTT) is a widely used operant task for measuring attention and motor impulsivity in rodents. Training animals in this task requires an extensive period of daily operant sessions. Recently, a self-paced, automated version of this task has been developed for mice, which substantially reduces training time. Whether a similar approach is effective for rats is currently unknown.

**Objective:**

Here, we tested whether attention and impulsivity can be assessed in rats with a self-paced version of the 5-CSRTT.

**Methods:**

Operant boxes were connected to home-cages with tunnels. Two groups of rats self-paced their training by means of an automated script. The first group of animals was allowed unlimited access (UA) to start trials in the task; for the second group, trial availability was restricted to the first 2.5 h of the dark cycle (TR). Task parameter manipulations, such as variable inter-trial intervals and stimulus durations as well as pharmacological challenges with scopolamine, were tested to validate the task.

**Results:**

Self-paced training took less than 1 week. Animals in the UA group showed higher levels of omissions compared with the TR group. In both protocols, variable inter-trial intervals increased impulsivity, and variable stimulus durations decreased attentional performance. Scopolamine affected cognitive performance in the TR group only.

**Conclusions:**

Home-cage-based training of the 5-CSRTT in rats, especially the TR protocol, presents a valid and fast alternative for measuring attention and impulsivity.

## Introduction

Animal models of executive functioning are pivotal to understanding the neurobiology of psychiatric illness. Executive function domains, such as attention and impulse control, are affected in several psychiatric disorders, including schizophrenia and attention-deficit hyperactivity disorder (ADHD) (Castellanos and Tannock 2002; Luck and Gold 2008). The 5-choice serial reaction time task (5-CSRTT) is a widely utilized behavioral paradigm for rodents to test visual sustained attention and motor impulsivity (Robbins 2002; Blondeau and Dellu-Hagedorn 2007). In this task, animals are trained to scan a horizontal array of 5 apertures for the onset of a visual stimulus and withhold responding until its appearance. After a stimulus presentation in one of the pseudo-randomly chosen apertures, the animal must make a response in the form of a nose poke within a limited time window. From typically 60 to 100 repetitions of these trials, attentional performance is deduced from the ratio of the number of correct and incorrect responses. Levels of motor impulsivity can be assessed from the number of premature responses before the onset of the visual cue. Importantly, possible non-specific effects of pharmacological or neuronal circuit interventions can be controlled for by assessing motor effects via different response latencies (Robbins 2002; Bari et al. 2008).

Before animals can perform this task reliably with a stimulus duration (SD) of typically 0.5 to 1.0 s, weeks to months of operant training are required (Bari et al. 2008; Bhandari et al. 2016). Not only is this labor-intensive, the long periods of food deprivation can add to the cumulative discomfort of animals during the experiment. Besides animal discomfort, idiosyncratic handling by the experimenter has been shown to alter behavioral outcomes in rats, such as learning and memory (Bohacek and Daniel 2007). Additionally, experimenter-induced interventions can increase corticosterone concentrations (Sorge et al. 2014; Deutsch-Feldman et al. 2015), which in turn could affect executive functioning (Sänger et al. 2014).

A previous study asserted the efficacy of a self-paced variant of the 5-CSRTT (SP-5-CSRTT) in mice. In that study, home-cages of animals were connected to operant 5-CSRTT chambers (the so-called CombiCage), and mice could self-pace task progression with minimal interference by experimenters (Remmelink et al. 2017). This adaptation of the 5-CSRTT led to a marked reduction in time that animals took to learn the task. Although the researchers reported slight differences in baseline performance at a SD of 1 s between animals trained in the SP-5-CSRTT and a conventional 5-CSRTT protocol, effects of behavioral challenges on attention and impulse control were similar. Additionally, the use of the SP-5-CSRTT for drug testing was shown by a dose-dependent effect of scopolamine, an acetylcholine muscarinic receptor antagonist, on attentive behavior (Remmelink et al. 2017). Whether this approach could be applied to testing attention and impulse control in rats is unknown. Additionally, whether task availability in the home-cage setting is an important factor for learning speed and performance is still unknown.

Here, we tested a modified version of the CombiCage SP-5-CSRTT, which was adjusted for rats. We measured training time and baseline performance and validated the SP-5-CSRTT by randomly varying behavioral parameters and quantifying effects on attention and impulsivity. Finally, we tested the effects of scopolamine, a muscarinic acetylcholine receptor antagonist, which has been shown to impact attention and impulsivity in rats in the conventional 5-CSRTT (Robbins 2002).

## Methods

### Animals

For training and testing in CombiCages, 36 male Long Evans rats (Janvier Labs, France, 8 weeks old) were initially housed in pairs with food and water available ad libitum 1 week before the start of experiments. Next, animals were housed individually in CombiCages, and behavioral procedures were initiated. Rats were housed under a 12-h light/dark cycle (lights off at 12 PM). For the conventional 5-CSRTT training, 14 male Long Evans rats (Janvier Labs, France, 8 weeks old) were housed individually. Food restriction began 1 week prior to behavioral training to achieve and maintain 85–90% of free feeding weight. Animals were trained daily for 5 days per week (Monday-Friday) as described in Luchicchi et al. (2016). One animal in the time-restricted (TR) group became sick after training and variable-ITI sessions and was excluded for the variable-SD session and scopolamine experiments. All experimental procedures were in accordance with the European and Dutch law and approved by the animal ethical care committee of the VU University and VU University Medical Center.

### SP-5-CSRTT task

**Fig. 1:**
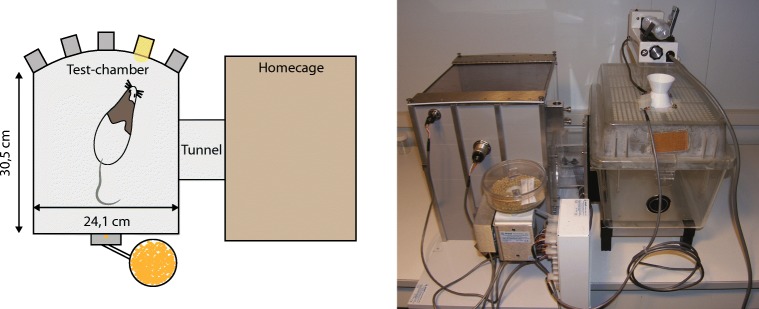
Schematic overview of the rat CombiCage. A standard macrolon home-cage is connected to a Med-Associates operant box by a polymer connection tube (diameter 10 cm). The operant box is equipped with a food magazine connected to a pellet dispenser. On the opposite wall five equally spaced cue holes are positioned with yellow LEDs. Each cue hole is equipped with an infrared response detector to measure nose-poke responses

For construction of CombiCages, a standard makrolon home-cage was connected to an operant box (Med-Associates Inc., St. Albans, VT, USA) with a custom-made polymer tube with a diameter of 10 cm. Operant chambers were on one side equipped with five cue holes, containing LED stimulus lights and infrared beam detectors. On the opposite wall, a food magazine, a red magazine light, and a yellow houselight were placed (Fig. 1).

Rats were placed in CombiCages 2 days before the experiment started, and food was available ad libitum. After the start of the task, animals earned their food in the form of pellets in the task (Dustless Precision Pellets, grain-based, F0165, 45 mg, Bio-Serve, USA). Animals were weighed each day before onset of the dark cycle. Animals were not food restricted prior to the start of the training. If rats did not earn enough pellets to gain weight according to an 85–90% food restriction regime, additional chow was given. In the present study, no additional chow was necessary during training, animals in the TR-group were fed extra chow after training to keep stable grow and performance during pharmacological testing.

For training in the SP-5-CSRTT, the same training stages were applied as in conventional 5-CSRTT training (Remmelink et al. 2017). First, animals learned to associate pellet delivery with reward during magazine training, and during 50 trials, a pellet was delivered after a variable inter-trial interval (ITI) of 4, 8, 16, or 32 s. Reward availability in the task was signaled by the magazine light, and collection of pellets triggered the next trial start. In the subsequent training stage, all five stimulus lights were lit until a nose-poke response was made in one of them to earn a reward. After 50 trials, animals moved on to the next stage. Here, a nose-poke response in the food magazine started an ITI period of 5 s followed by presentation of randomly selected single stimulus light. A nose poke into the lit cue hole was rewarded with a pellet; incorrect nose pokes were not punished.

In the next stage, rats started trials with a nose poke in the food magazine, starting an ITI of 5 s. Subsequently, one of the 5 cue holes was lit for a certain SD. Initially, SDs were 16 s and were titrated down in five steps to 1 s for the final stage. Rats had to make a response in the lit stimulus hole during stimulus presentation or within a 2-s limited hold period after stimulus presentation. A lack of response was considered an omission and resulted in a time-out period of 5 s. Incorrect and premature responses, during the ITI, also resulted in a time-out period of 5 s. Additionally, these errors were signaled with the houselight that was switched on for the duration of the time-out period. Correct responses were rewarded with a food pellet. After reward collection in the food magazine, rats could start the next trial 5 s later with a subsequent nose poke in the food magazine. We refer to the period of reward collection before start of the next trial as the “eat-interval.”

For the SP-5-CSRTT protocols, the performance criterion to reach a following stage with shorter SD was a minimum of 50 started trials with accuracy levels (ratio of correct and incorrect responses, see below) > 80% and either omissions < 20% or number of correct trials > 200 in the current stage. These parameters were calculated online during task performance using a sliding window of 20 trials on which accuracy levels and percentage omissions were calculated. This approach was based on recent work in mice (Remmelink et al. 2017). If the animal passed the performance criterion in this block of 20 trials analyzed by the sliding window, the program automatically moved to the next stage (Remmelink et al. 2017).

Two different groups were trained in CombiCages with different trial availabilities. In the unlimited access (UA) protocol, animals could initiate trials 24 h per day, whereas in the TR protocol, rats could only start trials during the first 2.5 h of the dark cycle. To examine effects of manipulation of task parameters, both groups were subjected to a session with variable ITIs (5, 7.5, or 12.5 s) or variable SDs (0.2, 0.5, or 1 s). These sessions comprised of a block of 500 trials for the UA group and a 2.5-h session for the TR group.

In the conventional 5-CSRTT group, rats were trained in the same training stages as described for the home-cage protocols. The criterion to move on to the next stage was set at accuracy > 80% and omissions < 20%. Performance was calculated after each half-hour session.

### Drug administration

Scopolamine hydrochloride (Sigma-Aldrich, St. Louis, MO, USA) was dissolved in 0.9% saline and injected intraperitoneally (i.p.) 20 min prior to the start of the dark phase. Scopolamine was freshly prepared on each test day, and doses were administered using a Latin square design on Monday, Wednesday, and Friday. Animals continued with training on Tuesday, Thursday, Saturday, and Sunday.

### Data analysis and statistics

All data were acquired with MED-PC software (Med-Associates, USA). Data analyses and statistics were done with custom-written scripts in MATLAB (Mathworks, USA). Accuracy was calculated a: (#correct) / ((#correct + #incorrect) ∗ 100). Omissions and premature responses were expressed as percentage of the total number of trials. Correct-response latency was expressed as the time in seconds between stimulus onset and a correct response. Magazine latency was expressed as the time in seconds between the correct response and pellet collection. Trials with a magazine latency > 10 s were excluded from further analysis. Normality of the data was tested with the Shapiro-Wilk test.

For comparison of training time and the number of required trials per training stage in the different groups, a Wilcoxon rank-sum test and two-way mixed repeated-measures ANOVA were used with group as between-subjects factor. Post hoc testing was performed using Wilcoxon rank-sum tests or *t* tests with Benjamin-Hochberg false discovery rate (FDR) to adjust *p* values for multiple comparisons (Benjamini and Hochberg 1995).

To compare baseline performance between groups, a block of 500 trials at SD1 for the UA group after passing SD1 criterion was compared with a 2.5-h session of SD1 trials for the TR group. Additionally, we compared baseline performance of the first 100 trials of the dark cycle for the TR and UA protocol with the CT baseline session. For both analyses, *t* tests or Wilcoxon rank-sum tests were performed.

Behavioral challenges with variable ITI or SD were only performed in the SP-5-CSRTT protocols and were analyzed using two-way mixed repeated-measures ANOVAs. To test differences in the number of started trials, accuracy, premature responses, and omissions between the dark period and light period for the UA group, *t* tests on grand means were performed.

The effect of scopolamine was tested in 2.5-h variable-ITI sessions (TR group), or data from the first 2.5 h in the dark cycle was analyzed (UA group). 2.5-h sessions were split in 30-min blocks for analyses. For the different behavioral parameters, two-way mixed repeated-measures ANOVAs were employed with dose and time as within-subject factors. Post hoc testing was performed with FDR-controlled *t* tests or Wilcoxon rank-sum tests.

In all cases, the significance level was set at *p* < 0.05.

## Results

### Training time is less than 1 week in SP-5-CSRTT

To test whether attention and impulsivity in rats can be assessed using an automated, self-paced task, as previously described for mice (Remmelink et al. 2017), we trained two groups of rats in an automated, modified home-cage version of the 5-CSRTT. Briefly, the home-cage of the animals was connected to an operant cage with a tunnel creating a CombiCage (Fig. 1). To test whether limited trial availability would increase motivation and affect learning speed and performance of animals, two protocols were tested that differed solely in trial availability. In the first protocol, the UA group could start trials throughout light and dark cycles for 24 h, whereas in the second protocol, the TR group could only start trials during the first 2.5 h of the dark cycle. Additionally, we have included data from a group of rats conventionally trained in the 5-CSRTT, by means of daily 30-min training sessions (Luchicchi et al. 2016). This data was included to show training and baseline performance of animals that were trained in a conventional 5-CSRTT in our lab.

**Fig. 2:**
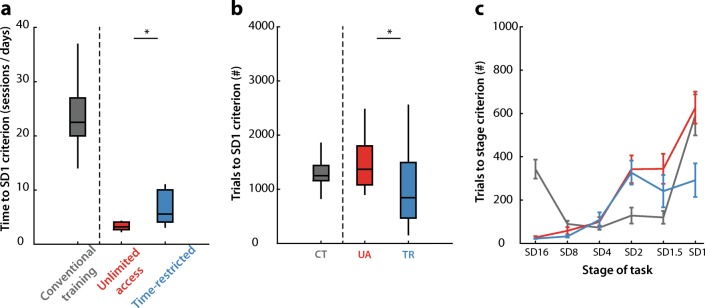
Training time to SD1 criterion performance is less than 1 week in home-cage 5CSRTT protocols. **a** Number of training sessions (conventional protocol) or training days (home-cage protocol) to SD1 criterion in the task. **b** Total number of trials to reach SD1 criterion. CT, conventional training; UA, unlimited access; TR, time-restricted. **c** Number of trials to reach criterion performance during each learning stage of the task for the different protocols. Data are expressed as mean ± SEM. *n* = 14 for conventional training (CT), *n* = 12 for both the unlimited access (UA), and time-restricted group (TR). * *p* < 0.01 Wilcoxon rank-sum test or *t* test between UA and TR protocol. Conventional training separated by vertical dashed line

Animals in both the UA and TR group were trained to SD 1 criterion in less than 7 days (Table 1, Fig. 2a). In particular, rats in the UA group finished training in less than 3.5 days and were quicker than the TR group (Fig. 2a; Wilcoxon rank-sum test, *p* < 0.01). However, the total number of trials that was required to finish SD1 to stable baseline performance criterion was less for the TR group (Fig. 2b; *t* test, *p* < 0.01). Closer inspection of the number of trials required per stage of the task did reveal differences in learning between the groups (Fig. 2c; group × stage: *F* [5,110] = 4.26, *p* < 0.01). Specifically, learning of the final stage, SD1, required less trials for the TR group compared with the UA protocol (Fig. 2c; FDR-corrected Wilcoxon rank-sum test, *p* < 0.01).

**Table 1:** Summary of training variables and performance for conventional training and both the unlimited access and time-restricted home-cage 5CSRTT groups

	Unlimited access	Time-restricted	Conventional training
Training			
Number of rats	12	12	14
Days to finish SD1 criterion	3.29 ± 0.76	6.55 ± 2.99	23.21 ± 6.13
Number of trials to SD1 criterion	1499 ± 522	1023 ± 717	1347 ± 409
Weight difference (% start vs end training)	+ 2 ± 2.8	+ 1 ± 1.9	NA
Earned pellets/day	282 ± 41	268 ± 29	60.71 ± 7.97
Punishment after error	TO + HL on	TO + HL on	TO + HL off
Eat-interval (s)	5	5	0
Performance at SD1			
Started trials per session/day (#)	832 ± 182	390 ± 60	100 ± 0
Accuracy (%)	83.11 ± 8.12	84.63 ± 4.32	84.61 ± 5.84
Omissions (%)	49.52 ± 8.14	20.03 ± 8.31	17.46 ± 7.65
Premature responses (%)	0.12 ± 0.08	0.32 ± 0.26	10.90 ± 5.14
Correct-response latency (s)	1.66 ± 0.29	1.41 ± 0.30	0.66 ± 0.36
Magazine latency (s)	2.21 ± 0.47	2.05 ± 0.41	1.51 ± 0.48
First 100 trials SD1 session			
Accuracy (%)	80.33 ± 11.69	83.55 ± 5.72	84.61 ± 5.84
Omissions (%)	51 ± 10.28	16.33 ± 8.42	17.46 ± 7.65
Premature responses (%)	0.33 ± 0.49	1.17 ± 1.53	10.90 ± 5.14
Correct-response latency (s)	1.67 ± 0.27	1.45 ± 0.34	0.66 ± 0.36
Magazine latency (s)	2.09 ± 0.42	2.11 ± 0.44	1.51 ± 0.48

*TO* = 5 s time-out; *HL* = houselight. Data are expressed as mean ± SD

### Stable baseline performance in the SP-5-CSRTT

**Fig. 3:**
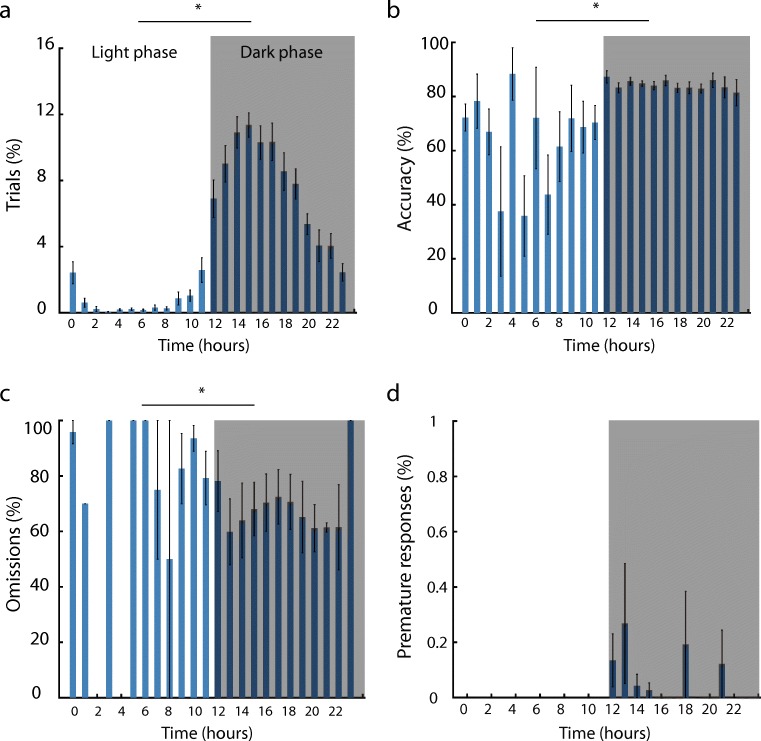
Behavioral performance over the light/dark cycle in the unlimited access group. **a**–**d** Performance in home-cage 5C unlimited protocol distributed over the day. Time is indicated as hour of the day, and time bins in shading represent the dark phase of the day. * *p* < 0.05 paired *t* test light vs dark phase. *n* = 12. Data are expressed as mean ± SEM

Similar to mice (Remmelink et al. 2017), in rats, the UA group also started significantly more trials during the dark phase of day-night cycles (91.1% of total) than during the light phase (Fig. 3a, *p* < 0.001). In addition, accurate responding was higher during the dark phase (Fig. 3b, *p* < 0.001), and omissions were lower, compared with the light phase (Fig. 3c, *p* = 0.018). Surprisingly, the percentage of premature responses, a measure for motor impulsivity, was below 1% of the number of trials. Levels of premature responding did not differ between the light or dark phase (Fig. 3d, *p* = 0.097). Since animals started trials almost exclusively during the dark phase and because of differences in task performance during the light and dark phase, we will henceforth only report behavioral parameters analyzed for trials started during the dark phase.

**Fig. 4:**
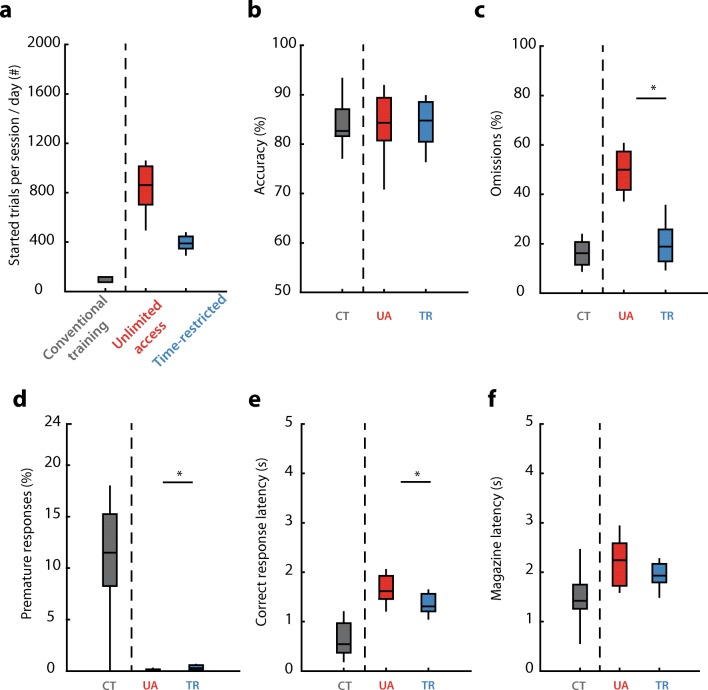
Behavioral performance at SD1 in the conventional 5CSRTT task and home-cage 5CSRTT protocols. **a** Number of started trials per session (conventional 5C) or per day (home-cage protocols). **b**–**f** Performance at SD1, data displayed for the measured task parameters. * *p* < 0.05 *t* test between UA and TR protocol. Conventional training separated by vertical dashed line. CT, conventional training; UA, unlimited access; TR, time-restricted. *n* = 14 for conventional training, *n* = 12 for each home-cage 5C group. Data are expressed as mean ± SEM

Next, we analyzed baseline SD1 performance across behavioral parameters and compared them between protocols (Table 1). The UA group rats started more than 800 trials per day on average, whereas the TR group started close to 400 trials (Fig. 4a). Accuracy, the measure for attention, did not differ between protocols (Fig. 4b, *t* test, *p* = 0.64), with rats in all groups reaching levels of approximately 85% correct choice at SD1. Interestingly, the percentage of omitted trials markedly differed between protocols (Fig. 4c, *t* test, *p* < 0.001). UA group rats showed almost three times more omissions than the TR group. Premature responding was reduced in the UA group compared with the TR protocol (Fig. 4d, *t* test, *p* < 0.05). Finally, whereas correct-response latencies were slightly elevated in the UA group compared with the TR protocol (Fig. 4e, *t* test, *p* = 0.04), magazine latencies were comparable (Fig. 4f, *t* test, *p* = 0.35). When we compared the first 100 trials of the UA and TR session, we only found significant differences in the percentage of omissions between the protocols (Table 1, Wilcoxon rank-sum test, *p* < 0.0001).

### SP-5-CSRTT performance is modulated by variable ITI and SD manipulations

**Fig. 5:**
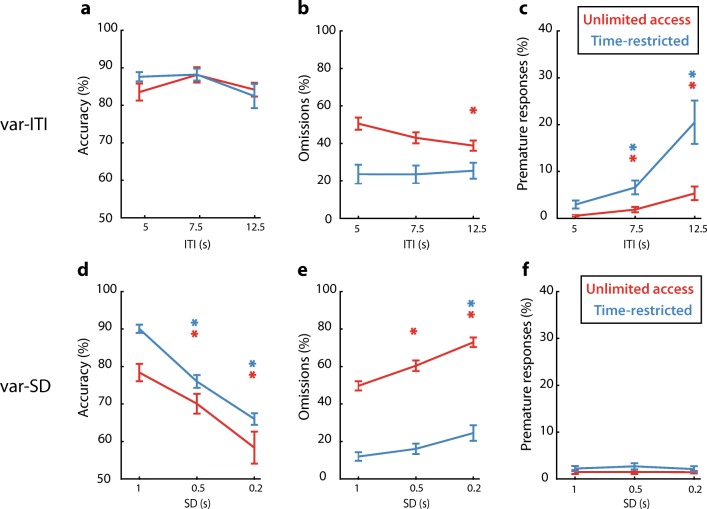
Behavioral performance in home-cage 5C is affected by varying SD and ITI. **a**–**c** Effect of varying the inter-trial interval (var-ITI) on selected task parameters. * *p* < 0.05 FDR-corrected paired *t* test vs ITI = 5 s. The color of the asterisk depicts in which group the difference is detected. **d**–**f** Effect of varying the stimulus duration (var-SD) on selected task parameters. * *p* < 0.05 FDR-corrected paired *t* test vs SD = 1 s. The color of the asterisk depicts which group the difference is detected. UA, unlimited access (*n* = 12); TR, time-restricted protocol (*n* = 11). Data are expressed as mean ± SEM

Since we observed differences in baseline performance in the SP-5-CSRTT compared with the conventional 5-CSRTT, we asked whether behavioral challenges would affect performance equally in the different protocols. For this, we subjected rats from the UA and TR group to days with either a variable ITI or a variable SD. Randomly varying the ITI between 5, 7.5, and 12.5 s affected accuracy to the same extent in both groups (Fig. 5a, ITI: *F* [2,42] = 5.18, *p* < 0.01; group × ITI: *F* [2,22] = 1.97, *p* = 0.15). However, post hoc testing revealed no significant differences in accuracy between trials with different ITI durations. The percentage of omitted trials was significantly decreased for the UA group on trials with the longest ITI (Fig. 5b, ITI: *F* [2,42] = 6.57, *p* < 0.01; group × ITI: *F* [2,22] = 10.08, *p* < 0.001). Premature responses were significantly and differentially increased in the UA and TR group (Fig. 5c, ITI: *F* [2,42] = 23.42, *p* < 0.001; group × ITI: *F* [2,22] = 8.3, *p* < 0.001), with the TR group showing the strongest increase in premature responding at the longest ITI.

Variable SDs between 1, 0.5, and 0.2 s significantly affected accuracy to the same extent in both protocols, with a decrease at shorter SDs (Fig. 5d, SD: *F* [2,42] = 68.45, *p* < 0.001; group × SD: *F* [2,22], *p* = 0.3). Omissions were differentially increased in the groups, with increments in the UA group at 0.5 and 0.2 s, whereas only the shortest SD increased omissions in the TR group (Fig. 5e, SD: *F* [2,42] = 81.34, *p* < 0.001; SD × group: *F* [2,22] = 7.14, *p* < 0.001). Premature responses were not affected in either group by varying the SD (Fig. 5f, SD: *F* [2,42] = 0.57, *p* = 0.57; SD × group: *F* [2,22] = 0.42, *p* = 0.66). Taken together, these data show that varying ITIs mainly affected premature responding in both groups, with subtle effects on omissions in the UA group, whereas variable SD conditions caused decrements in accuracy and increments in omissions in both UA and TR group, similar to what has been reported previously in the conventional 5-CSRTT (Robbins 2002; Bari et al. 2008).

### Effects of scopolamine on behavioral performance

During the scopolamine experiments, one animal in the TR group and one animal in the UA group did not start trials after the high dose (0.3 mg/kg) and were therefore excluded from analyses. For further pharmacological validation of the SP-5-CSRTT protocols, we used scopolamine, a muscarinic acetylcholine receptor antagonist. Scopolamine has previously been shown to affect multiple aspects of executive functioning in both the conventional 5-CSRTT in rats, as well as the automated home-cage 5-CSRTT in mice (Pattij et al. 2007; Hodges et al. 2009; Remmelink et al. 2017). The 2.5-h session of the TR group was analyzed in five 30-min blocks considering the short half-life of scopolamine in rats (Lyeth et al. 1992). For the UA group, we analyzed the first 2.5 h in 30-min blocks.

**Fig. 6:**
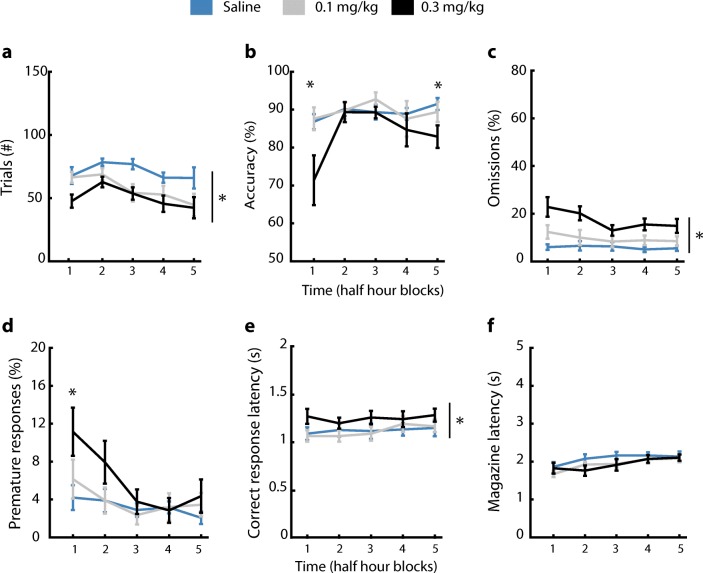
Scopolamine affects cognitive parameters in time-restricted (TR) home-cage 5C task. **a** Scopolamine decreased the number of started trials over the session. * *p* < 0.05 main effect dose repeated-measures ANOVA. **b** Accuracy was reduced by the highest dose of scopolamine in the first and fifth time block. * *p* < 0.05 FDR-corrected Wilcoxon signed-rank test 0.3 mg/kg vs saline. **c** Scopolamine increased the percentage of omitted trials over the session. * *p* < 0.05. **d** Scopolamine increased the percentage of premature responses in the first time block of the session. *p < 0.05 FDR-corrected Wilcoxon signed-rank test 0.3 mg/kg vs saline. **e** Correct-response latency was increased after scopolamine administration. * *p* < 0.05 main effect dose repeated-measures ANOVA. **f** Scopolamine did not affect the magazine latency. *n* = 10. Data are expressed as mean ± SEM

In the TR SP-5-CSRTT protocol, scopolamine decreased the number of started trials throughout the 2.5-h session and to a similar extent across the 30-min blocks. The number of started trials also decreased over time in a session (Fig. 6a, dose: *F* [2,9] = 18.44, *p* < 0.001, time: *F* [4,9] = 5.37, *p* < 0.01, dose × time: *F* [8,9] = 1.06, *p* = 0.4). The high dose of scopolamine, 0.3 mg/kg, decreased accuracy of responding in the first and last half hour block of the session (Fig. 6b, dose: *F* [2,9] = 4.88, *p* < 0.05, time: *F* [4,9] = 5.59, *p* < 0.01, dose × time: *F* [8,9] = 2.56, *p* < 0.05). Omissions were dose-dependently increased by scopolamine throughout the entire session (Fig. 6c, dose: *F* [2,9] = 13.59, *p* < 0.001, time: *F* [4,9] = 4.06, *p* < 0.01, dose × time: *F* [8,9] = 1.65, *p* = 0.13). Scopolamine specifically increased premature responses during the first half hour block at the highest dose, and overall premature responding decreased over time (Fig. 6d, dose: *F* [2,9] = 2.25, *p* = 0.13, time: *F* [4,9] = 9.26, *p* < 0.001, dose × time: *F* [8,9] = 2.5, *p* < 0.05). Correct-response latencies were increased by scopolamine throughout the session (Fig. 6e, dose: *F* [2,9] = 6.72, *p* < 0.01, time: *F* [4,9] = 2.87, *p* < 0.05, dose × time: *F* [8,9] = 1.87, *p* = 0.08). Magazine latencies were not affected by administration of scopolamine, but increased over the half hour time blocks (Fig. 6f, dose: *F* [2,9] = 1.71, *p* = 0.21, time: *F* [4,9] = 10.2, *p* < 0.001, dose × time: *F* [8,9] = 1.09, *p* = 0.38). In conclusion, scopolamine affected attention and impulse control performance in the TR SP-5-CSRTT similarly as has been reported previously for the conventional 5-CSRTT and SP-5-CSRTT in mice (Pattij et al. 2007; Hodges et al. 2009; Remmelink et al. 2017).

**Fig. 7:**
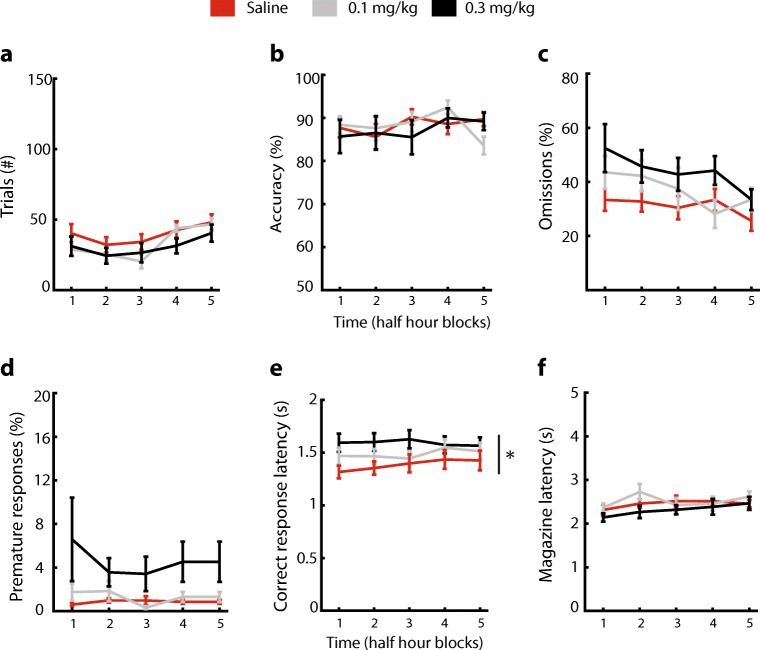
Scopolamine does not affect attention and inhibitory control in unlimited access (UA) home-cage 5C task. **a**–**f** Effect of scopolamine on selected task parameters. Scopolamine increased the correct-response latency but did not alter other parameters. * *p* < 0.05 main effect dose repeated-measures ANOVA. *n* = 11. Data are expressed as mean ± SEM

In the UA SP-5-CSRTT protocol, scopolamine did not affect the number of started trials, but this variable was affected by time (Fig. 7a, dose: *F* [2,10] = 1.33, *p* = 0.29, time: *F* [4,10] = 8.87, *p* < 0.001, dose × time: *F* [8,10] = 0.82, *p* = 0.59). Accurate responding was not affected by scopolamine administration or by time (Fig. 7b, dose: *F* [2,10] = 0.11, *p* = 0.9, time: *F* [4,10] = 1.19, *p* = 0.33, dose × time: *F* [8,10] = 1.01, *p* = 0.43). Scopolamine did not alter omissions in the task, which were affected by time (Fig. 7c, dose: *F* [2,10] = 2.13, *p* = 0.15, time: *F* [4,10] = 5.4, *p* < 0.01, dose × time: *F* [8,10] = 1.49, *p* = 0.18). Premature responses were not affected by either scopolamine or time (Fig. 7d, dose: *F* [2,10] = 3.23, *p* = 0.06, time: *F* [4,10] = 0.94, *p* = 0.45, dose × time: *F* [8,10] = 0.77, *p* = 0.63). Scopolamine increased correct-response latencies throughout the session (Fig. 7e, dose: *F* [2,10] = 4.31, *p* < 0.05, time: *F* [4,10] = 0.81, dose × time: *F* [8,10] = 1.17, *p* = 0.33). Finally, magazine latencies were not altered by scopolamine administration (Fig. 7f, dose: *F* [2,10] = 1.51, *p* = 0.25, time: *F* [4,10] = 1.95, *p* = 0.13, dose × time: *F* [8,10] = 1, *p* = 0.44). In summary, scopolamine failed to affect attention and inhibitory control in the UA SP-5-CSRTT protocol but increased correct-response latencies.

## Discussion

We present an automated home-cage-based version of the 5-CSRTT for rats, as was previously developed for mice (Remmelink et al. 2017). Our main findings are that in the SP-5-CSRTT, training time was less than 1 week and that animals gained weight during training without the necessity of prior food restriction. SP-5-CSRTT was sensitive to behavioral challenges in similar fashion as demonstrated in the conventional 5-CSRTT, whereas only in the TR-group, pharmacological interventions with scopolamine were effective.

Conventional 5-CSRTT requires long training periods (Granon et al. 2000; Hahn and Shoaib 2002; Bari et al. 2008; Luchicchi et al. 2016). Rats with UA to SP-5-CSRTT finished training in less than 4 days, while rats with TR access finished within 1 week. The training time reduction most likely results from the increased number of trials that rats performed each day. Interestingly, the total number of trials to reach SD1 criterion was reduced for the TR protocol. A closer look at the number of required trials per stage revealed that learning dynamics differed between protocols. Rats trained in the TR protocol required less trials to learn the final stage under SD1 conditions. One factor contributing to this different rate of learning could be the continuous food availability in the UA protocol. This might increase satiety and decrease motivation, possibly reflected by the increase in percentage omissions as discussed below.

Baseline performance in SP-5-CSRTT differed on several parameters between protocols. Rats in the UA group started trials preferably in the dark phase compared with the light phase (Loos et al. 2014; Remmelink et al. 2017; Rivalan et al. 2017). Omissions were dramatically increased in the UA group, possibly resulting from reduced motivation or reduced salience of visual cues in light surroundings. Restriction of trial accessibility (TR) strongly reduced levels of omissions in the SP-5-CSRTT. In human subjects, similar observations have been made regarding time limits in motivation and task performance. When subjects were given twice the necessary amount of time needed for solving an addition task, it not only took longer to complete the task, but easier task goals were set (Locke 1968). To our knowledge, our study is the first to directly compare effects of time limits on task performance in rodents. In addition, levels of premature responding were lower in both home-cage 5-CSRTT protocols. In mice, no differences in levels of premature responding or increases in omissions were reported between conventional training and home-cage 5-CSRTT (Remmelink et al. 2017). This might be due to subtle differences in task design, such as a longer eat-ITI in the mouse SP-5-CSRTT protocol or to inherent differences in premature responding strategies between mice and rats as previously reported (Young et al. 2013; Cope et al. 2016).

A potential caveat could be differences in signaling of response errors in the tasks. In the conventional 5-CSRTT, error are punished by time-out periods signaled through house-light extinction (Bari et al. 2008). In SP-5-CSRTT, time-out periods were signaled by turning on the house-light. Despite this, behavioral challenges, such as varying ITI or SD, resulted in similar effects to conventional 5-CSRTT (Bari et al. 2008; Saund et al. 2017; Schippers et al. 2017). Varying the ITI led to increased premature responding and decreased omissions. Increasing ITI durations has previously been reported to lower omissions in rats (Schippers et al. 2017), yet increases in omissions have also been shown (Chudasama et al. 2003; Saund et al. 2017). This may result from shorter limited hold periods urging faster responses following stimulus presentation. Shortening SDs decreased accurate choice as well as increased the percentage of omitted trials (Bari et al. 2008; Counotte et al. 2011). Thus in SP-5-CSRTT, variable ITIs mainly affected impulsive responding, while variable SDs mainly affected attentional performance. The validity of the SP-5-CSRTT for drug screening was demonstrated by scopolamine (muscarinic acetylcholine receptor antagonist) challenges, which have been well characterized in both mice and rats in conventional 5-CSRTT (Pattij et al. 2007; Hodges et al. 2009; Remmelink et al. 2017). Reported effects of scopolamine on attention and inhibitory control were replicated in the TR protocol, but not in the UA protocol. Scopolamine decreased accuracy mainly in the first half hour block of the TR protocol. Additionally, premature responding was specifically increased in the first half hour, in line with plasma half-life of scopolamine (Lyeth et al. 1992). In contrast, the number of started trials, correct-response latency, and omissions were affected throughout the entire session. Whereas in mice, scopolamine has been shown to robustly decrease accurate responding, in rats, results are inconsistent in literature (Pattij et al. 2007; Remmelink et al. 2017). Similar to our findings, decrements in accuracy have been reported (Jones and Higgins 1995; Mirza and Stolerman 2000). In contrast, several other studies found no effect of scopolamine on accurate choice in rats (Jäkälä et al. 1992; Higgs et al. 2000; Hodges et al. 2009). Interestingly, effects of scopolamine on accurate choice were mainly found under more challenging conditions, such as white noise distraction (Jones and Higgins 1995), or by reducing SDs (Mirza and Stolerman 2000). In the present study, animals were tested with long, variable ITI sessions to increase task unpredictability. Possibly, scopolamine affects attentional parameters when the cognitive load is increased. Alternatively, scopolamine effects may be rat strain-dependent, similar to effects of nicotine in the 5-CSRTT (Mirza and Bright 2001).

In the TR protocol, scopolamine also increased omission rate and decreased number of started trials throughout the entire 2.5-h duration of the session, suggesting decreased motivation. This could indicate that scopolamine impacts cognitive functions for a short period after injection, whereas its effects on motivation are longer-lasting. Reduced motivation may also be the main effect of scopolamine in the UA protocol, where we only found an increase in correct-response latency and no effect on accurate choice and premature responding. This finding is in contrast with the results in the TR protocol in rats and findings in mice where scopolamine did affect impulsivity and attentional processes in the SP-5-CSRTT (Remmelink et al. 2017). We hypothesize that the lower number of started trials in a specific time bin and the higher level of omissions in the UA protocol reflect diminished engagement in the task as mentioned above. This would make the UA protocol less valid for pharmacological testing than conventional 5-CSRTT or the TR protocol, especially for drugs with a short half-life like scopolamine. Careful consideration for the selection of training and testing protocol is thus necessary based on the research question.

One remaining question is how the SP-5-CSRTT contributes to habitual versus goal-directed responding in this task compared with conventional training. Learning of this task is based on reinforcement and continuation of similar task contingencies after reaching criterion performance results in stimulus-response habits or overtraining (Jog et al. 1999). This habitual form of responding lacks signs of cognitive contributions and exhibits insensitivity to value of the outcome and to changes in action-outcome contingencies (Yin and Knowlton 2006; Smith and Graybiel 2016). To our knowledge, the transition from goal-directed behavior to habitual responding has not directly been studied in the 5-CSRTT, by, for instance, changing action-outcome contingencies, i.e., by rewarding only 50% of correct responses and assess effects on performance. Since the SP-5-CRTT protocols allow the animals to perform more trials per day, they will potentially overtrain more quickly in the task. We therefore recommend that testing of pharmacological compounds takes place in cognitively challenging sessions, which require the animal to break fixed response routines.

A potential caveat of the SP-5-CSRTT is that rats were housed individually in CombiCages. Social isolation in rats can lead to increased stress levels and altered neuroendocrine state, particularly during early weaning (Serra et al. 2000; Weiss et al. 2004; Weintraub et al. 2010), which has been found to impact executive functions in rats (Kirkpatrick et al. 2013; Wang et al. 2017). Notably, these effects are most pronounced when social isolation occurs following early weaning, for instance starting at postnatal day 21. Our SP-5CSRTT training started when animals were at least 63 days old. It has recently been shown that prolonged individual housing of adult rats did not influence corticosterone concentration, hippocampal long-term potentiation measurements, and object place recognition (Riga et al. 2017). Combined with the restricted amount of experimental time, self-paced training, and less food restriction, stress effects are most likely limited in SP-5CSRTT. Social housing and home-cage testing can be combined in rats (Rivalan et al. 2017) and are important points of improvement of the SP-5-CSRTT. Secondly, the accelerated learning rate and format of the task might influence the neurobiological correlates of behavioral performance when compared with the conventional 5-CSRTT. Nevertheless, home-cage-based training of rats in the SP-5-CSRTT provides a rapid and reliable alternative for conventional training in the task to measure attention and motor impulsivity. The short training time opens up new possibilities and allows, for instance, specific testing of young or adolescent rats, which in the conventional paradigm is not possible due to time constraints. Thereby, SP-5CSRTT is highly suited to address questions involving pharmacological challenges or to investigate the physiological mechanisms of attention and motor impulsivity during limited time windows.
